# Salivary Alterations in Rats with Experimental Chronic Kidney Disease

**DOI:** 10.1371/journal.pone.0148742

**Published:** 2016-02-09

**Authors:** Ana Carolina Romero, Cassia Toledo Bergamaschi, Douglas Nesadal de Souza, Fernando Neves Nogueira

**Affiliations:** 1 Departamento de Biomateriais e Biologia Oral, Faculdade de Odontologia, Universidade de São Paulo, São Paulo, Brasil; 2 Departamento de Fisiologia, Escola Paulista de Medicina – UNIFESP, São Paulo, Brasil; Mario Negri Institute for Pharmacological Research and Azienda Ospedaliera Ospedali Riuniti di Bergamo, ITALY

## Abstract

**Objective:**

This study aimed to analyze changes in saliva composition and salivary secretion process of rats with chronic kidney disease induced by 5/6 nephrectomy to set the foundation for salivary studies related to CKD.

**Methods:**

CKD was induced in Wistar rats via 5/6 nephrectomy. Blood and saliva samples were collected from Control, Sham and CKD groups at 8 and 12 weeks after the surgery. Salivation was stimulated via intraperitoneal injections of pilocarpine (1.0 mg/Kg body weight) or isoproterenol (5.0 mg/Kg body weight). Saliva was collected and immediately stored at -80°C until analysis. The salivary flow rate, total protein, amylase and peroxidase activities, and urea concentrations were measured. The blood urea nitrogen (BUN) and serum creatinine concentrations were also evaluated.

**Results:**

Increases in BUN and serum creatinine concentrations were observed in the CKD groups. Amylase activity was significantly reduced in response to both stimuli in the CKD groups at 8 weeks and increased in the CKD groups at 12 weeks in response to isoproterenol stimulus. The peroxidase activities of the CKD groups were significantly reduced in response to isoproterenol stimulation and were increased at 12 weeks in response to pilocarpine stimulation. Salivary urea was significantly increased in the CKD groups at 8 weeks in response to the isoproterenol stimuli and at 12 weeks in response to both salivary agonists.

**Conclusions:**

The pattern of alterations observed in this experimental model is similar to those observed in patients and clearly demonstrates the viability of 5/6 nephrectomy as an experimental model in future studies to understand the alterations in salivary compositions and in salivary glands that are elicited by CKD.

## Introduction

Chronic kidney disease (CKD) is a major problem in public health worldwide [1, 2]. CKD is a progressive disorder marked by a loss of kidney function over time. The early stages of CKD are characterized by kidney damage and are generally asymptomatic. As the kidney disease worsens, kidney function begins to deteriorate, leading to end-stage kidney disease, which requires kidney transplantation or hemodialysis [3].

Main oral manifestations of patients with chronic renal failure include odor of urea, dry mouth, taste alterations, pain in the tongue and mucosa, gingivitis, periodontitis and a high prevalence of dental calculus [4–7].

Saliva is an essential fluid in the oral cavity, being its chemical composition closely associated to major functions contributing to the maintenance of oral health. Studies of salivary alterations in patients undergoing hemodialysis have reported decreases in stimulated and unstimulated salivary flow [8, 9], increases in the pH and buffering capacity [8], increases in protein secretion [9, 10], decreases in peroxidase enzymatic activity [9, 11], increases in the amylase concentration [12] and changes in the secretion of electrolytes associated with the increased secretions of phosphate, potassium, zinc, magnesium and urea [12–14]. Studies of the accessory salivary glands of CKD patients have reported decreases in the function of these glands, as determined by scintigraphy [15]; DNA damage, as observed with comet assays [16]; and atrophy and fibrosis observed via histological analyses [17].

Several experimental models of CKD have been used in studies with rats. Surgical renal mass ablation, antibody induced-nephritis and renal artery branch ligation are the most commonly used methods [18]. The 5/6 nephrectomy procedure consists of the ligation of two branches of the left renal artery and total right nephrectomy and results in the progressive decline of renal function. This method has been used in several studies to evaluate the systemic effects of CDK, such as cardiovascular alterations, proteinuria, changes in the liver function and Ca and P metabolism, and anemia [19–21]. Despite this wealth of studies, salivary alterations occurring in this experimental model of CKD in rats have not yet been analyzed.

This study aimed to determine the most significant changes in saliva composition and salivary secretion process that typify rats with chronic kidney disease induced by 5/6 nephrectomy, to establish the fundamentals for salivary studies related to CKD.

## Materials and Methods

### Animals

All animals were handled in accordance with the guidelines of Ethical Principles of Animal Experimentation adopted by COBEA. The protocol of this study was approved by the *Ethical Committee for Animal Research* of the Institute of Biomedical Sciences of the University of São Paulo.

Eight-week old male Wistar rats were group-housed in individual cages in climate-controlled conditions (22°C, 45–65% humidity, 12-h artificial light/dark cycle, noise level <55 dB) with free access to standard rodent food (Purina, Brazil) and water. The animals were divided into two main groups according to the experimental periods of 8 and 12 weeks. For each time period, the groups were divided into the following 3 subgroups: Control, Sham, and Chronic Kidney Disease (CKD). These subgroups were further divided into 2 groups according to whether they received an isoproterenol or pilocarpine salivary stimulus. The final numbers of animals in each group ranged from 8 to 10.

To induce CKD, a single phased 5/6 nephrectomy surgery was performed. The animals were anesthetized with ketamine (100 mg/kg/bw) and xylazine (10 mg/kg/bw), ventral position placed on a surgical table, shaved and disinfected with 70% ethanol. The effects of anesthesia were verified and complemented during the procedures. A ventral midline incision was made to expose the left kidney hilum. The left renal artery was carefully dissected, and two of the three branches were ligated with silk 6.0 lines, supported by a surgical microscope (Zeiss). The right renal hilum was ligated in order to perform total nephrectomy on the right kidney [19, 21, 22].

The results of the CKD groups were compared with those of the control (normal) and sham animals (i.e., animal submitted to the same surgical procedure without artery ligation and nephrectomy). The experimental periods of 8 and 12 weeks were recorded from the day of surgery and separation of the control animals.

### Sample collection

Blood and saliva samples were collected from all of the subgroups in both the 8- and 12-week experimental period groups.

Under the effect of anesthesia (ketamine and xylazine), salivation was stimulated by an intraperitoneal injection of pilocarpine (1.0 mg/Kg body weight) or isoproterenol (5.0 mg/Kg body weight), both of which were dissolved in distilled water, and the saliva that was produced was dripped in plastic tubes and maintained on ice during all the saliva collection period (40 minutes) [23]. The salivary flow was determined, and the samples were centrifuged at 1,540 x g at 4°C for 5 minutes. The supernatants were stored at -80°C for later analyses. Blood samples were collected from the heart, stored in tubes containing EDTA (15%), centrifuged at 1,540 x g for 10 minutes, and the sera refrigerated until analyses.

### Analysis

Protein concentration (mg of protein/mL of saliva) was measured using Folin phenol reagent as described by Lowry [24] and having bovine serum albumin as protein standard. Sample absorbances were measured at 660 nm in a Beckman DU-800 spectrophotometer (Beckman, Fullerton, CA).

Amylase activity (mg of maltose/mg of salivary protein) was determined by the method described by Fisher and Stein [25] using a solution of maltose as standard. The color intensity was measured at 530 nm on a Beckman DU-800 spectrophotometer (Beckman, Fullerton, CA).

Peroxidase activity was determined using Chandra’s method [26], as modified by Anderson [27]. Interference due to pseudoperoxidase activity was eliminated by performing duplicate assays in the presence of 10 mM 3-amino-1, 2 triazole, which is an inhibitor of peroxidase activity. The absorbance was measured at 460 nm in a Beckman DU-800 spectrophotometer (Beckman, Fullerton, CA). Concentrations of peroxidase in the samples are expressed in μg of peroxidase/mg of protein, and these values were based on a standard curve constructed with different concentrations of lactoperoxidase.

The Urea CE Kit (Labtest, Brazil) was used to determine blood urea nitrogen (BUN) and urea concentrations in saliva. The developed color intensity was measured at 600 nm in a DU-800 spectrophotometer (Beckman, Fullerton, CA), and a standard containing 70 mg/dL was used to calculate the concentrations of the samples. The results are expressed as mg urea/dL of blood or saliva.

Serum creatinine concentration was measured using a Labtest kit (Labtest). Samples were analyzed at 520 nm in a DU-800 spectrophotometer (Beckman, Fullerton, CA). A standard containing 4 mg/dL was used to calculate the unknown concentrations. The results are expressed in mg creatinine/dL of blood or saliva.

All results were statistically analyzed with analysis of variance (ANOVA) and Tukey's contrast tests, with an assumed level of significance of 5%. Pearson correlation analyses between BUN and salivary urea results were also conducted using Minitab 16 software.

## Results

No significant differences were observed in the initial and final body weights of the 3 experimental groups at 8 or 12 weeks. Weekly water consumption was 50% higher in the CKD group after 8 weeks (p<0.05) and remained over 50% higher until 12 weeks (p<0.05) after surgery (Table 1). However, food intake did not change during these time periods (data not shown).

**Table 1:** Weekly consumption of water from Control, Sham, and chronic kidney disease (CKD) groups measured at 2, 4, 8 and 12 weeks of experiment.

Groups	Weeks			
	2	4	8	12
**Control**	237 ± 23 (n = 16)	210 ± 17 (n = 16)	223 ± 18 (n = 16)	214 ± 19 (n = 8)
**Sham**	208 ± 14 (n = 16)	200 ± 12 (n = 16)	233 ± 19 (n = 16)	219 ± 25 (n = 8)
**CKD**	277 ± 46* ^#^ (n = 16)	313 ± 51* ^#^ (n = 16)	388 ± 38* ^#^ (n = 16)	358 ± 93* ^#^ (n = 8)

Data (mean ± SD) expressed in milliliters. n = number of animals.

The * indicates differences from Control and ^#^ differences from Sham group (p<0.05), on the same experimental period.

The presence of chronic kidney disease is demonstrated in Table 2. Significant increases in BUN concentrations were observed at 8 and 12 weeks in the CKD groups compared to Sham (138% and 88%, respectively) and Control (130% and 93%, respectively) groups. Significant increases in serum creatinine were also observed in CKD animals at 8 weeks compared with Sham (82%) and Control (35%) groups and at 12 weeks compared with Sham (63%) and Control (50%) group.

**Table 2:** BUN and creatinine concentrations of the Control, Sham and chronic kidney disease (CKD) groups at 8 and 12 weeks of experiment.

	8 weeks			12 weeks		
	Control	Sham	CKD	Control	Sham	CKD
**BUN (mg/dL)**	30.5 ± 3.78 (n = 19)	29.73 ± 2.06 (n = 16)	70.1 ± 14.76 * ^#^ (n = 15)	30.85 ± 3.75 (n = 19)	32.1 ± 4.34 (n = 19)	60.07 ± 17.2 * ^#^ (n = 17)
**Creatinine (mg/dL)**	1.01 ± 0.4 (n = 19)	0.75 ± 0.12 (n = 16)	1.37 ± 0.35 * ^#^ (n = 15)	0.74 ± 0.23 (n = 19)	0.68 ± 0.13 (n = 19)	1.11 ± 0.29 * ^#^ (n = 17)

Values are expressed in mean ± SD. n = number of animals.

The * indicates differences from Control and ^#^ differences from Sham group (p<0.05), on the same experimental period.

The salivary flow rate, total protein concentration, amylase activity, peroxidase activity and salivary urea results from the Control, Sham and CKD groups following stimulation with pilocarpine or isoproterenol are presented in Tables 3 and 4, respectively.

**Table 3:** Analysis of saliva stimulated by Pilocarpine (1mg/Kg of b.w.) of Control, Sham and chronic kidney disease (CKD) groups at 8 and 12 weeks of experiment.

	8 weeks			12 weeks		
	Control	Sham	CKD	Control	Sham	CKD
**Flow rate (μL/min)**	47.8 ± 11.4 (n = 9)	54.4 ± 15.5 (n = 8)	59.3 ± 14.8 (n = 9)	39.0 ± 10.6 (n = 10)	33.0 ± 9.5 (n = 9)	34.7 ± 13.0 (n = 8)
**Protein (mg/ml)**	0.98 ± 0.19 (n = 8)	0.90 ± 0.24 (n = 8)	1.04 ± 0.27 (n = 8)	1.25 ± 0.32 (n = 10)	1.42 ± 0.53 (n = 9)	2.56 ± 2.13 * (n = 8)
**Amylase (mg of maltose/mg protein)**	331 ± 80 (n = 9)	436 ± 100 (n = 7)	240 ± 142 ^#^ (n = 7)	273 ± 84 (n = 10)	234 ± 68 (n = 9)	211 ± 133 (n = 8)
**Peroxidase (μg/mg protein)**	1.47 ± 0.54 (n = 8)	1.41 ± 0.66 (n = 8)	1.50 ± 0.80 (n = 7)	5.14 ± 3.14 (n = 10)	5.96 ± 3.23 (n = 9)	46.4 ± 34.9 * ^#^ (n = 8)
**Salivary urea (mg/dL)**	51.47 ± 7.5 (n = 8)	51.47 ± 6.2 (n = 8)	105.1±25.67 * ^#^ (n = 8)	42.86 ± 4.9 (n = 7)	45.98 ± 6.51 (n = 7)	93.49 ± 20.7 * ^#^ (n = 8)

Values are expressed in mean ± SD. n = number of animals.

The * indicates differences from Control and ^#^ differences from Sham group (p<0.05), on the same experimental period.

**Table 4:** Analysis of saliva stimulated by Isoproterenol (5mg/Kg of b.w.) of Control, Sham and chronic kidney disease (CKD) groups at 8 and 12 weeks of experiment.

	8 weeks			12 weeks		
	Control	Sham	CKD	Control	Sham	CKD
**Flow rate (μL/min)**	7.52 ± 2.93 (n = 10)	7.65 ± 2.05 (n = 8)	6.04 ± 2.32 (n = 8)	10 ± 3.9 (n = 9)	13 ± 4.22 (n = 10)	6.68 ± 3.58 ^#^ (n = 9)
**Protein (mg/ml)**	32.8 ± 2.5 (n = 10)	34.62 ± 2.13 (n = 8)	39.71 ± 7.29 (n = 8)	51.63 ± 4.8 (n = 7)	41.04 ± 6.94 (n = 10)	42.24 ± 4.55 * (n = 7)
**Amylase (mg of maltose/mg protein)**	5.37 ± 2.25 (n = 10)	4.12 ± 1.25 (n = 8)	3.03 ± 0.76 * ^#^ (n = 8)	3.84 ± 0.76 (n = 7)	2.97 ± 0.56 (n = 10)	7.15 ± 5.8 * ^#^ (n = 7)
**Peroxidase (μg/mg protein)**	26.29 ±6.16 (n = 10)	27.32 ± 6.15 (n = 8)	12.62 ± 3.02 * ^#^ (n = 8)	19.26 ± 3.0 (n = 7)	18.47 ± 6.7 (n = 10)	11.36 ± 1.5 * ^#^ (n = 7)
**Salivary urea (mg/dL)**	49.74 ± 9.84 (n = 10)	42.84 ± 5.02 (n = 8)	147.96 ± 23.92 * ^#^ (n = 8)	50.7 ± 7.45 (n = 7)	57.62 ± 9.1 (n = 10)	174.5± 93 * ^#^ (n = 7)

Values are expressed in mean ± SD. n = number of animals.

The * indicates differences from Control and ^#^ differences from Sham group (p<0.05), on the same experimental period.

**Fig 1:**
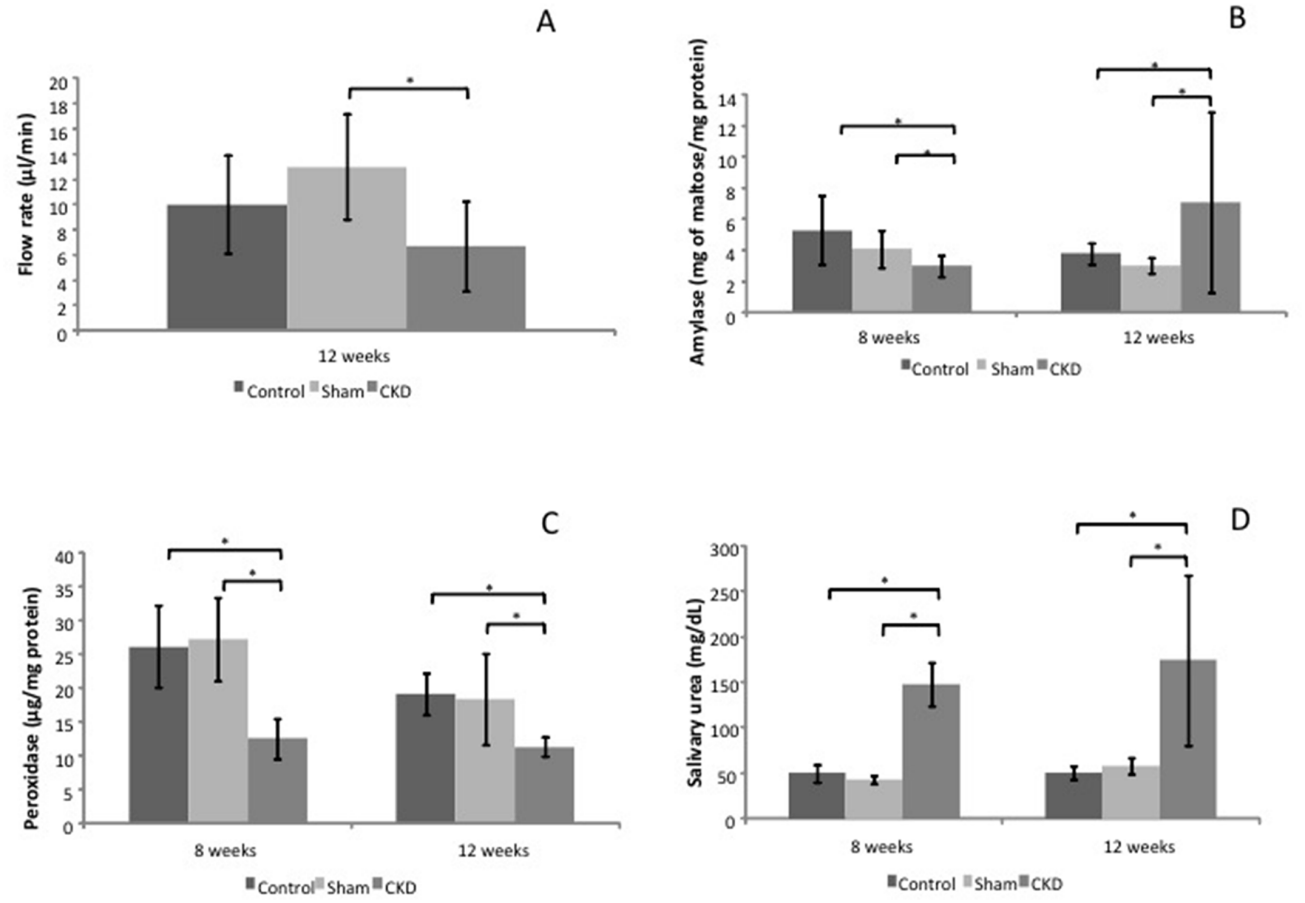
Representative graphics of analyzed saliva stimulated by isoproterenol (5mg/Kg of b.w.). (A) Salivary flow rate from Control, Sham and CKD groups at the experimental period of 12 weeks. (B) Salivary amylase activities from Control, Sham and CKD groups at the experimental periods of 8 and 12 weeks. (C) Salivary peroxidase activities from Control, Sham and CKD groups at the experimental periods of 8 and 12 weeks. (D) Salivary urea concentrations from Control, Sham and CKD groups at the experimental periods of 8 and 12 weeks. * indicates significant differences between indicated groups (p< 0.05).

The flow rate was not affected with pilocarpine stimulation at 8 or 12 weeks (Table 3). However, isoproterenol stimulation elicited significant decreases at 12 weeks (49%) compared with the Sham group (Fig 1A).

**Fig 2:**
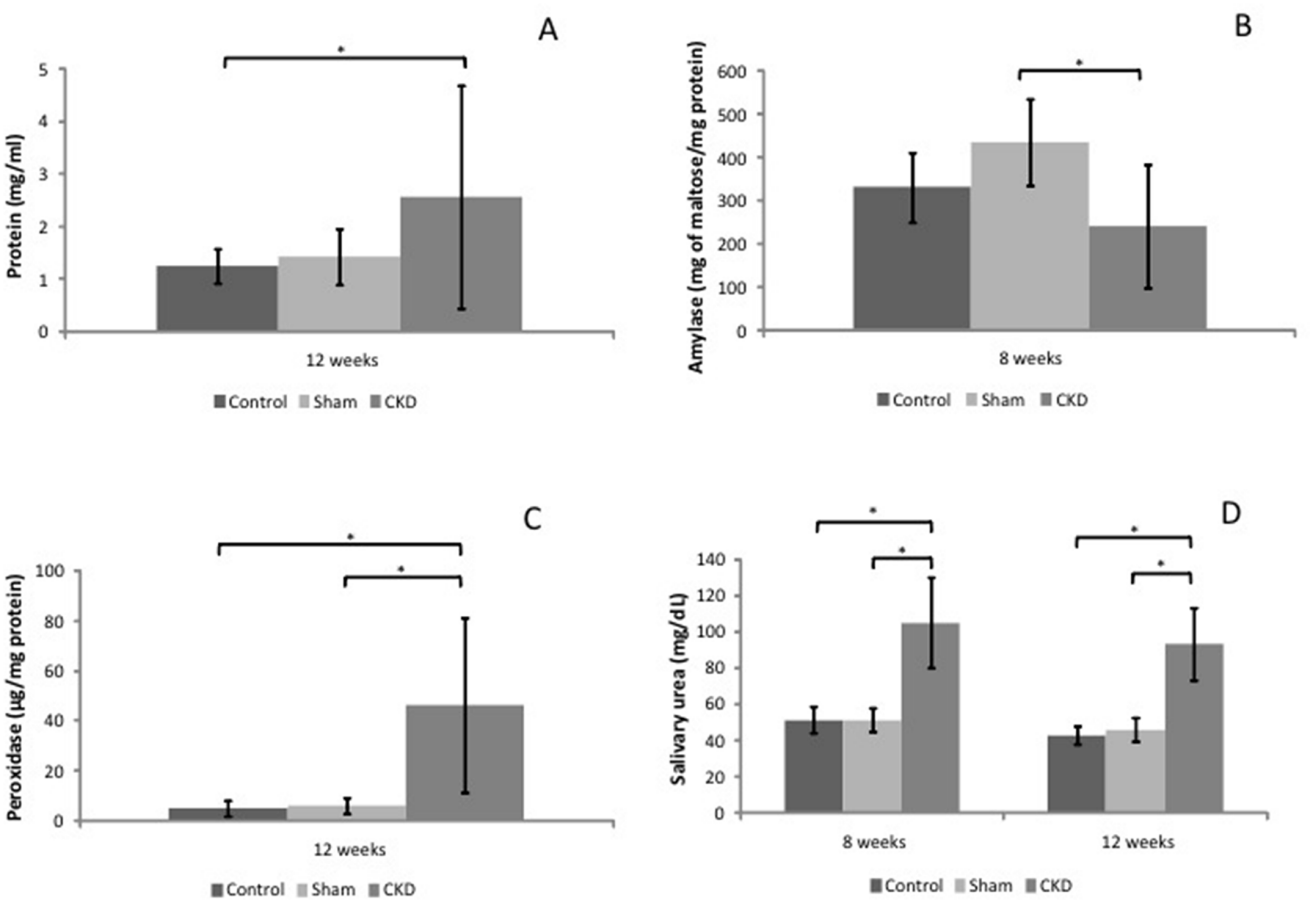
Representative graphics of analyzed saliva stimulated by pilocarpine (1mg/Kg of b.w.). (A) Salivary total protein concentrations from Control, Sham and CKD groups at the experimental period of 12 weeks. (B) Salivary amylase activities from Control, Sham and CKD groups at the experimental periods of 8 weeks. (C) Salivary peroxidase activities from Control, Sham and CKD groups at the experimental period of 12 weeks. (D) Salivary urea concentrations from Control, Sham and CKD groups at the experimental periods of 8 and 12 weeks. * indicates significant differences between indicated groups (p< 0.05).

Changes in total protein concentrations were observed only 12 weeks after surgery, with a significant increase in response to pilocarpine stimulation (107%; Fig 2A), and a significant decrease (29%) in response to isoproterenol stimulation, compared with the control animals.

The enzymatic activities of amylase and peroxidase were affected in the CKD groups. At 8 weeks, amylase activity was reduced in the CKD group in response to the isoproterenol stimulus compared with the Sham (27%) and Control (44%) animals (Fig 1C). Pilocarpine stimulus elicited a reduction in amylase activity of 45% compared with the Sham animals (Fig 2B). However, at 12 weeks, amylase activity was significantly increased in the CKD group in response to isoproterenol stimulus compared with the Sham (140%) and Control (86%) groups (Fig 1B).

Significant reductions in peroxidase activity were observed in the saliva samples collected after isoproterenol stimulation 8 weeks after surgery (approximately 50% compared with the Sham and Control animals) and 12 weeks after surgery (approximately 40% compared with the Sham and Control animals; Fig 1C). The pilocarpine stimulus elicited a large increase in peroxidase activity in the CKD group 12 weeks after surgery (approximately 900% compared with the Sham and Control animals; Fig 2C).

Salivary urea concentrations were significantly increased in the CKD groups at 8 and 12 weeks in response to the pilocarpine (over 100%) (Fig 2D) and isoproterenol (over 200%) stimuli compared with the Control and Sham groups (Fig 1D).

**Fig 3:**
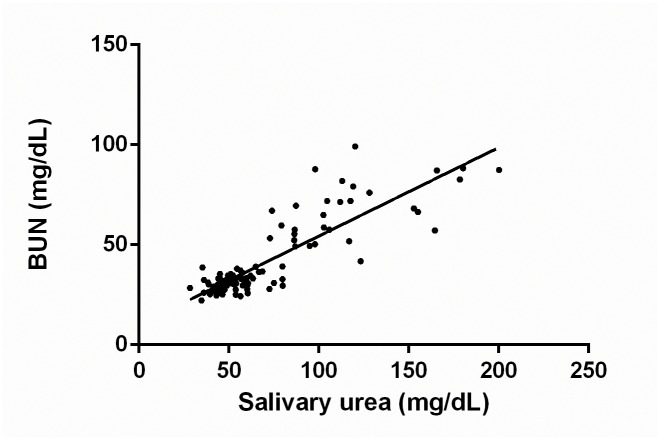
Correlation between BUN and salivary urea concentrations. n = 105. Data were expressed in mg/dL.

Fig 3 demonstrates the correlation between BUN and salivary urea concentrations including all groups, salivary agonists, and experimental times (Pearson’s correlation R = 0.779, R^2^ = 0.607, p<0.01).

## Discussion

Saliva is an important fluid in the oral cavity. The salivary composition is closely related to its functions, which are important for the maintenance of oral health [28]. The salivary composition and flow rate are dependent on several factors, such as circadian rhythms, light exposure, degree of hydration, stimulus type, age and sex [29–31], and on the presence of systemic disorders like chronic kidney disease.

To our knowledge, this is the first work that sought to determine the effects of 5/6 nephrectomy, an experimental model for the study of CKD, on such a plethora of salivary parameters. The difficulty to collect sufficient volume of in rest non-invasive saliva in rats restricts the extrapolation of the present findings to a human non-pharmacologically manipulated clinical condition, although clearly shows the pattern of secretion and salivary composition in response to parasympathetic and sympathetic stimuli in rats with CKD.

The chosen model was the 5/6 nephrectomy, which causes renal function deficiencies due to the long-term structural and functional adaptations of the remaining kidney tissue. However, during the experiment, renal function declined, causing systemic complications, such as uremia [21, 32]. Some authors have described the experimental period of 8 weeks as a moderate stage of CKD (reductions in renal function of approximately 50%) [19]. The experimental period of 12 weeks was chosen to study the same parameters following a longer exposure.

The animals of this study were feed *ad libitum* with a standard chow, with casein as the protein source. One of the recommendations for controlling the progression of CKD is restricting the dietary protein consumption [33]. A reduction or manipulation of the quality of the protein source intake (e.g. soy) could influence the salivary changes with a slower or faster progression of CKD, and changes in overall diet composition can also promote changes in salivary composition [34, 35].

Rats are widely used as experimental animals in studies of salivary secretion and salivary gland morphology in several disorders. This animal was chosen due to the similarity of its salivary glands to those of humans, particularly regarding the structural organization and the secretion products [36]. Studies monitoring the salivary pharmacokinetic changes induced by drugs, such as procainamide and ofloxacin, in rats with chronic renal impairment have been reported [37, 38], but there are no studies that have used nephrectomy models to analyze the salivary composition parameters and their correlations with serum changes resulting from the disease.

In experimental models, salivary secretion can be stimulated by drugs. Pilocarpine has been used as a parasympathetic agonist whilst isoproterenol has been used as a sympathetic agonist. Sympathetic stimulation acts on the β-adrenergic receptors in salivary glands, resulting in increased intracellular cAMP concentrations that activate protein kinase A (PKA), which results in the production of saliva with a higher protein content and lower water volume. In turn, parasympathetic stimulation acts on muscarinic cholinergic receptors to primarily activate phospholipase C, which subsequently causes an increase in the intracellular concentration of calcium that leads to the secretion of great amounts of water and electrolytes. Under parasympathetic stimuli, saliva secretion is induced in response to the activation of protein kinase C (PKC) and complex formation of Ca^2+^-calmodulin, a mechanism less efficient in protein secretion than the one responsible for saliva secretion under sympathetic stimulation, thus resulting in the production of fluid saliva with lower protein content and higher amount of water and electrolytes [28, 39].

There is no consensus about the chronic kidney disease effects in the salivary flow. Several authors have previously demonstrated that there are no changes in salivary flow in different stages of the disease [12, 40], on the other hand, other studies found significant decreases, especially in patients undergoing hemodialysis, related to direct glandular damage and/or restriction in fluid intake [17, 41]. In this study, the 12 weeks CKD animals exhibited significant decreases of salivary flow comparing with Sham group under isoproterenol stimulus. Some authors reported autonomic nervous system dysfunction in patients in pre-dialysis and dialysis and also a hyperactivity of the sympathetic nervous system in uremia with decreased responsiveness of α- and β-adrenergic receptors, due to the chronic increase in sympathetic stimulation [42, 43]. Thus, the lower responsiveness of glandular receptors could be responsible for such decrease in salivary flow, but further studies are needed to confirm this hypothesis.

Amylase accounts for approximately 50% of the total protein produced by the salivary glands [44–46]. It is a metalloenzyme that catalyzes the hydrolysis of glycosidic linkages in polysaccharides, such as starch. Amylase is also important for the formation of the acquired enamel pellicle and bacteria adhesion to the tooth surface. A significant increase in salivary amylase activity was observed in CKD group in response to isoproterenol stimulation 12 weeks after surgery. Hyperamylasemia is a common finding in patients with chronic renal failure. Some authors have reported increases in serum, pancreatic and salivary amylase concentrations in patients with varying degrees of chronic kidney disease and patients on hemodialysis [12, 47]. However, the clinical significance of these findings remains unknown. Several authors have unsuccessfully attempted to use the amylase activity level as a marker of pancreatic damage in CKD. Although there is a consensus regarding the presence of hyperamylasemia in CKD, it has not yet been possible to establish a numerical correlation between amylase activity and disease stage. The observed high amylase concentration in saliva of animals with CKD confirms the previously observed clinical findings in patients.

Peroxidase is an enzyme with antimicrobial properties that catalyzes the oxidation of thiocyanate (SCN^-^) in the presence of hydrogen peroxide (H_2_O_2_) to generate hypothiocyanate (OSCN^-^), which inhibits bacterial growth, prevents the accumulation of this toxic substance, and acts as an antioxidant enzyme [48]. At 8 weeks, decreased renal function led to a significant decrease in peroxidase activity after isoproterenol stimulation. At 12 weeks, significant changes in the activity of this enzyme were observed in the CKD group. Previous studies reported oxidative stress in uremia, increased production of reactive oxygen species and decreased antioxidant system activity in patients with chronic renal failure [49, 50]. Oxidative stress is a potential mediator of cardiovascular, neurological, and inflammatory diseases related to chronic renal failure [51]. The increase in peroxidase activity suggests that the changes in the antioxidant systems of the salivary glands in the CKD group were the result of increased production of free radicals in the disease.

Increases in serum creatinine and BUN concentrations are used to evaluate the presence and severity of chronic kidney disease [20, 50]. In our study, after 8 weeks, these parameters increased in the CKD group, and these increases remained constant after 12 weeks of the experiment, relative to the control and sham groups; these findings are indicative of increased retention of these compounds in response to reductions in the renal function in these animals. The observed improvement in renal function at 12 weeks comparing with 8 weeks CKD animals can be related with the rats’ malnourishment as a consequence of the renal failure itself, with underproduction of creatinine, in the absence of a better method to estimate the glomerular filtration rate. An increase in water consumption was also observed in the CKD group, but no difference was seen in terms of food consumption. Polydipsia and polyuria are symptoms of CKD and result from the body’s attempts to eliminate excess accumulations of toxic substances.

The increased salivary urea concentration observed in the CKD animals was strongly and positively correlated with the plasma concentration, which confirms the findings of clinical studies that have demonstrated the potential of using saliva to identify markers of the disease. The use of saliva as a diagnostic tool has been highly recommended because saliva collection is less invasive than blood collection and consequently more readily accepted by patients [52, 53].

In this study, we analyzed the effects of 5/6 nephrectomy on the composition of stimulated saliva collected from rats with the intention of using this experimental animal model in future studies of salivary gland dysfunction. The alterations observed in the saliva are very similar to those that have been detected in patients, clearly demonstrating the viability of the 5/6 nephrectomy animal model as an excellent experimental model for future studies that seek to understand whether changes in salivary composition and salivary glands are promoted by CKD.
